# Comparative analysis of soil properties before and after *Morchella sextelata* cultivation across various soil types

**DOI:** 10.3389/fmicb.2025.1700246

**Published:** 2025-10-28

**Authors:** Juan Zhao, Rui Zeng, Chengming Zhang, Bin He, Qin Zhang, Qihong Zhou, Zikang Gong, Honglin Liu, Songqing Liu

**Affiliations:** ^1^Sichuan Provincial Key Laboratory for Development and Utilization of Characteristic Horticultural Biological Resources, College of Chemistry and Life Sciences, Chengdu Normal University, Chengdu, China; ^2^Meishan Vocational & Technical College, Meishan, China

**Keywords:** morel (*Morchella sextelata*), soil, physicochemical property, metagenomics, microbial community

## Abstract

*Morchella*, a highly nutritious edible fungus, has been successfully cultivated through artificial means. However, as cultivation areas have expanded, declining yield have emerged more prominently. Soil physicochemical characteristics and microbial communities were critical to production on cultivating morels. In this study, our results reveals that cultivation significantly alters soil properties and microbial communities in a soil type-dependent manner. In sandy soil, pH and key nutrients (total nitrogen, total phosphorus, available phosphorus) increased, while potassium and calcium levels decreased. Microbial diversity decreased in sandy soil but increased in paddy soil, with the overall community structure in sandy soil being more drastically reshaped. Metagenomic profiling identified distinct differential taxa and functional shifts, showing that sandy soil exhibited greater enrichment of microbial genes, including soil-borne diseases. These findings demonstrate that *M. sextelata* cultivation induces considerable and contrasting changes in soil nutrient profiles and microbiome composition, with sandy soil being more susceptible to microbial restructuring and potential pathogen enrichment.

## 1 Introduction

Morels (*Morchella* spp.) are edible fungi highly valued for their exceptional nutritional content and distinctive flavor, offering considerable economic and research significance (Deng et al., 2021). Widely used in culinary and medicinal applications, morels are recognized for their unique aroma, rich nutrient profile, and high levels of essential amino acids and organic compounds (Wu et al., 2021). Morel cultivation relies on exogenous nutrition bag (ENB) technology to supply nutrients for developing mature morel fruiting bodies, which is the critical step for large-scale morel cultivation (Liu et al., 2023a; Xie G. et al., 2024). In Sichuan Province, the scale of artificial morel cultivation has steadily expanded, resulting in increased income for local growers (Xu et al., 2022). However, this rapid growth has also introduced several challenges now affecting production, including disease outbreaks and continuous cropping obstacles (Guo et al., 2016; He et al., 2017).

Recent studies suggest that shifts in soil nutrient availability and microbial community composition are major contributors to declining yield (Xu et al., 2024). It reported that nutrient accumulation following morel cultivation can lead to reduced yields (Tan et al., 2019). Nitrogen (N) and phosphorus (P) accumulation and potassium (K) loss are identified as the main factors responsible for the yield decline in continuous cropping. Additionally, *Morchella*, acting as an invasive species, substantially decreases both the richness and evenness of soil fungal communities during cultivation. Continuous cropping also lead to an increased dominance of specific fungal taxa, like *Mortierella*, *Solicoccozyma*, *Humicola* and *Trichocladium*, which increased soil nutrients to inhibit differentiation (Tan et al., 2021a; Wei-Ye et al., 2022; Zhang et al., 2023).

However, the selection of suitable soil still largely depends on growers’ production experience due to the limited availability of scientific guidance. In field, sandy soil are the common soil types for morel cultivation. Recently, rice–morel rotation has emerged as an efficient agricultural model increasingly promoted in southern China. This system seeks to maximize the use of dormant winter rice fields, thereby improving land utilization and increasing farmers’ income. Studies have also shown that rice–morel rotation combined with a 10% nitrogen reduction holds strong potential for enhancing overall nitrogen use efficiency and improving rice growth performance (Song et al., 2025). Additional research suggests that this rotation system contributes to improved soil health (Duan et al., 2023). Therefore, rice–morel rotation warrants further in-depth investigation and broader application.

In this study, we investigated changes in soil physicochemical properties and microbial communities in both sandy and paddy soils before and after morel cultivation, with the goal of identifying suitable soil environments for optimal growth. This research aims to provide theoretical guidance for improving morel cultivation practices and advancing the rotational cropping system of rice and morel.

## 2 Materials and methods

### 2.1 Soil samples collection

Soil samples were collected in March 2024 from Meishan, Sichuan, China, where morels (*Morchella sextelata*) were cultivated either in sandy soil or paddy soil previously used for rice cultivation. We collected soil samples after the morel harvest. The “before morel cultivation” soil samples were obtained from an uncultivated area within the same designated plot. The sandy soil, which defined as soil characterized by a high sand content and properties of being loose and well-aerated, was used for morel cultivation for the first time. While the paddy soil, which defined as soil characterized by a heavy, clay-rich texture and high water retention capacity, had grown rice before but was also newly used for morels—neither field experienced continuous cropping. Paddy soil before or after addition of with *M. sextelata* was abbreviated as RCK and RTM, respectively. Sandy soil before or after addition of with *M. sextelata* was abbreviated as SCK and STM, respectively (Table 1).

**Table 1:** Sample abbreviations and descriptions used in this study.

Sample abbreviation	Sample description
CK	Soil before addition of with *M. sextelata*
TM	Soil after adding *M. sextelata*
RCK	Paddy soil before addition of with *M. sextelata*
RTM	Paddy soil after adding *M. sextelata*
SCK	Sandy soil before addition of with *M. sextelata*
STM	Sandy soil after adding *M. sextelata*

Cultivation followed a standardized technical protocol (Liu et al., 2023b). Rhizosphere soil samples were collected using the five-point sampling method. At each point, soil cores were taken from three depths (0, 5, and 10 cm), and three replicates were obtained per depth to form a composite sample. Fifteen soil cores were taken and thoroughly mixed to form a single composite sample. This composite was then divided into five replicates for analysis. Each replicate was further split into two portions: one portion was air-dried in the shade for physicochemical analysis, while the other was flash-frozen in liquid nitrogen and stored at −80 °C for metagenomic sequencing.

### 2.2 Soil physicochemical property analysis

Soil physicochemical properties were measured in accordance with established standards (Wei-Ye et al., 2022). The following methods were used: pH value (NY/T 1121.2-2006); total nitrogen (NY/T 53-1987); total potassium (LY/T 1234-2018); available potassium (NY/T 889-2004); total phosphorus (LY/T 1232-2015); available phosphorus (NY/T 1121.7-2014); total calcium (HJ781-2016); and exchangeable calcium (NY/T 1121.13-2016). A *t*-test was applied to analyze the results of soil physicochemical properties.

### 2.3 DNA extraction and sequencing

According to previously described methods, total genomic DNA was extracted from soil samples using the CTAB method (Tanase et al., 2015). The DNA was randomly fragmented into ~350 bp segments using a Covaris ultrasonic disruptor. Library construction involved end repair, A-tailing, adapter ligation, purification, and PCR amplification. After quality assessment, the libraries were subjected to paired-end 150 bp (PE150) sequencing.

### 2.4 Metagenomic analysis

We used Fastp to filter raw sequencing data and obtain clean reads for gene prediction and abundance analysis. Assembly was performed using Bowtie2 (Karlsson et al., 2013). Open reading frames were predicted with MetaGeneMark, and redundant sequences were removed using the cluster database at high identity with tolerance (CD-HIT) (Li and Godzik, 2006; Mende et al., 2012; Li et al., 2014). Clean reads were then aligned to the nonredundant gene catalog using Bowtie2 (Qin et al., 2010). Based on gene abundance in the catalog, we conducted basic statistical analysis, core-pan gene analysis, and correlation analysis and generated Venn diagrams of gene numbers. For taxonomic annotation, unigenes were aligned to the Micro_NR database using DIAMOND. This database includes bacterial, fungal, archaeal, and viral sequences extracted from the National Center for Biotechnology Information nonredundant (NR) database (Buchfink et al., 2015).

### 2.5 Alpha and beta diversity analysis

Alpha diversity indices, including Shannon and Simpson, were calculated using quantitative insights into microbial ecology (Caporaso et al., 2010). Based on lowest common ancestor annotation and the gene abundance table, we determined the taxonomic abundance for each sample along with the corresponding gene abundance tables (Feng et al., 2015). We then generated a relative abundance overview and an abundance clustering heatmap, followed by dimensionality reduction analyses using PCA and nonmetric multidimensional scaling (NMDS) (Le Chatelier et al., 2013; Stewart et al., 2014).

### 2.6 Functional analysis

MetaGenomeSeq was used to perform permutation tests between groups at each taxonomic level, generating corresponding *p*-values. LEfSe analysis was conducted using LEfSe software, with the default linear discriminant analysis (LDA) score threshold set to 4 (Le Chatelier et al., 2013). DIAMOND software was used to align unigenes against the KEGG, CAZy and PHI databases for functional annotation (Cantarel et al., 2009; Kanehisa et al., 2017; Urban et al., 2025).

## 3 Results

### 3.1 Physicochemical property analysis of *Morchella sextelata*-cultivation soil

**Figure 1:**
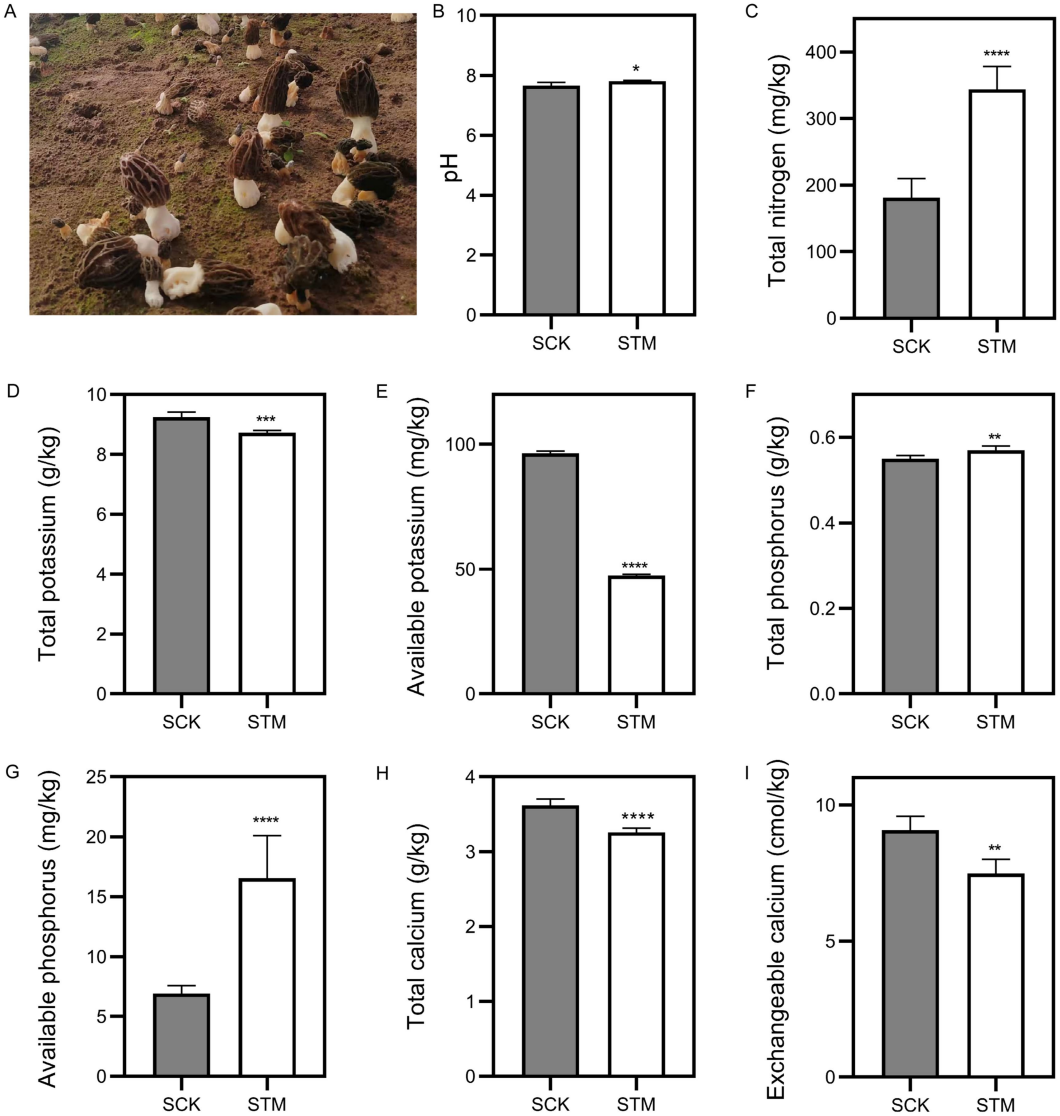
Physicochemical properties of sandy soil. **(A)** Morel fructification of the sandy soil used in this study. Measurement of **(B)** pH, **(C)** total nitrogen, **(D)** total potassium, **(E)** available potassium, **(F)** total phosphorus, **(G)** available phosphorus, **(H)** total calcium, and **(I)** exchangeable calcium in sandy soil. Data were analyzed using Student’s *t*-test: **p < 0.01, ***p < 0.001, and ****p < 0.0001.

**Figure 2:**
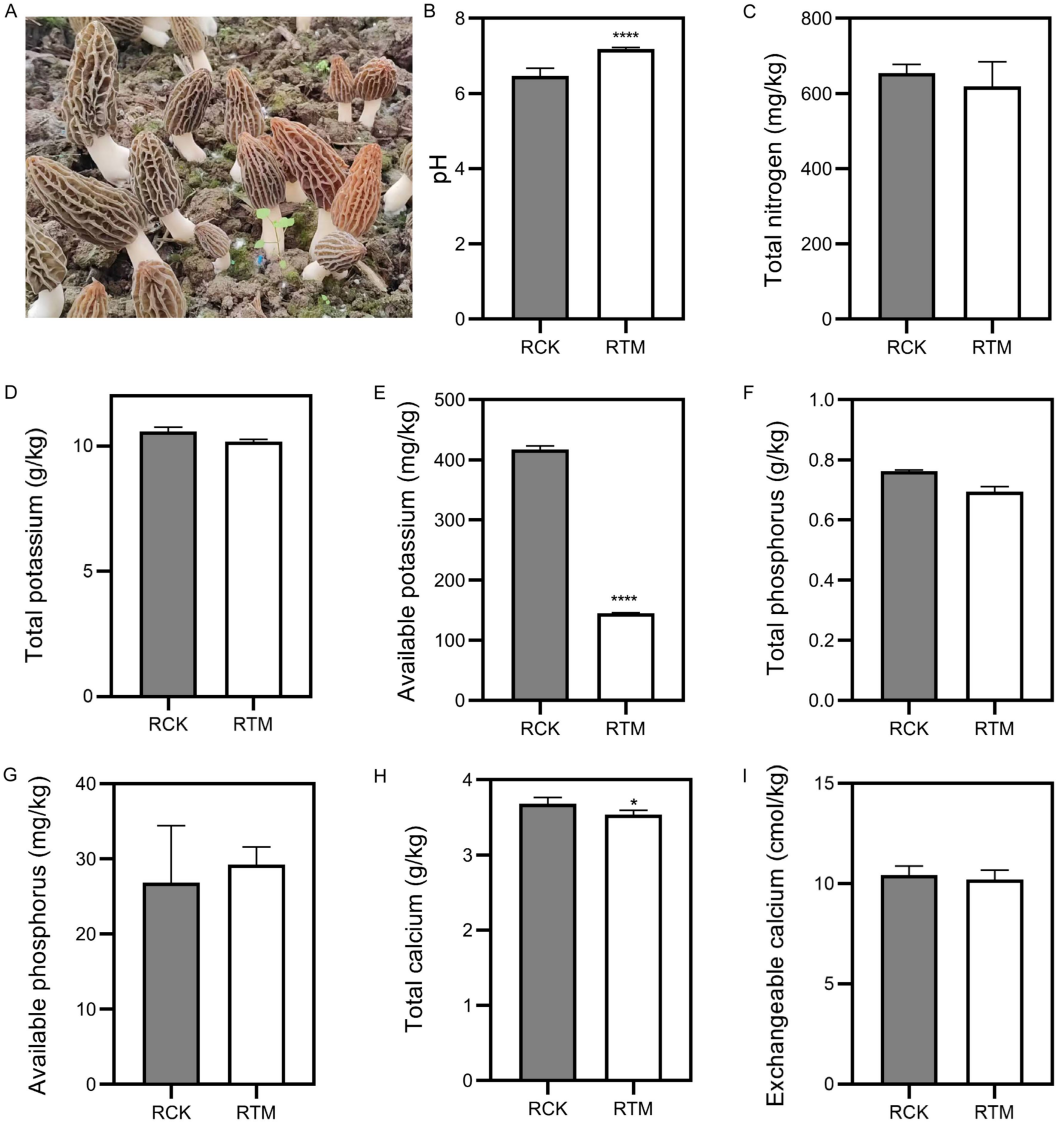
Physicochemical properties of paddy soil. **(A)** Morel fructification of paddy soil used in this study. Measurement of **(B)** pH, **(C)** total nitrogen, **(D)** total potassium, **(E)** available potassium, **(F)** total phosphorus, **(G)** available phosphorus, **(H)** total calcium, and **(I)** exchangeable calcium in paddy soil. Data were analyzed using Student’s *t*-test; *p < 0.05 and ***p < 0.0001.

To examine the differences between paddy and sandy soils, we collected samples before and after *M. sextelata* cultivation. Based on soil physicochemical property standards, we measured soil pH and nutrient content. Results indicated pH, total nitrogen (N), total phosphorus (P), and available phosphorus significantly increased about 1.9, 47.7, 3.6 and 139%, respectively (Figure 1). However, total potassium (K), available K, total calcium (Ca), and exchangeable Ca showed significant decreases in sandy soil about 5.8, 50.8, 9.9 and 17.6%, respectively. In paddy soil, the pH is significantly evaluated about 12.1%, and total Ca and available K levels dropped substantially about 3.8 and 78%, respectively (Figure 2). Additionally, no significant changes (*p* > 0.05) were observed in the other nutrients in paddy and sandy soil. Overall, these findings suggest that *M. sextelata* cultivation led to considerable alterations in the nutrient profile of sandy soil.

### 3.2 Functional analysis of soil microbiome composition

**Figure 3:**
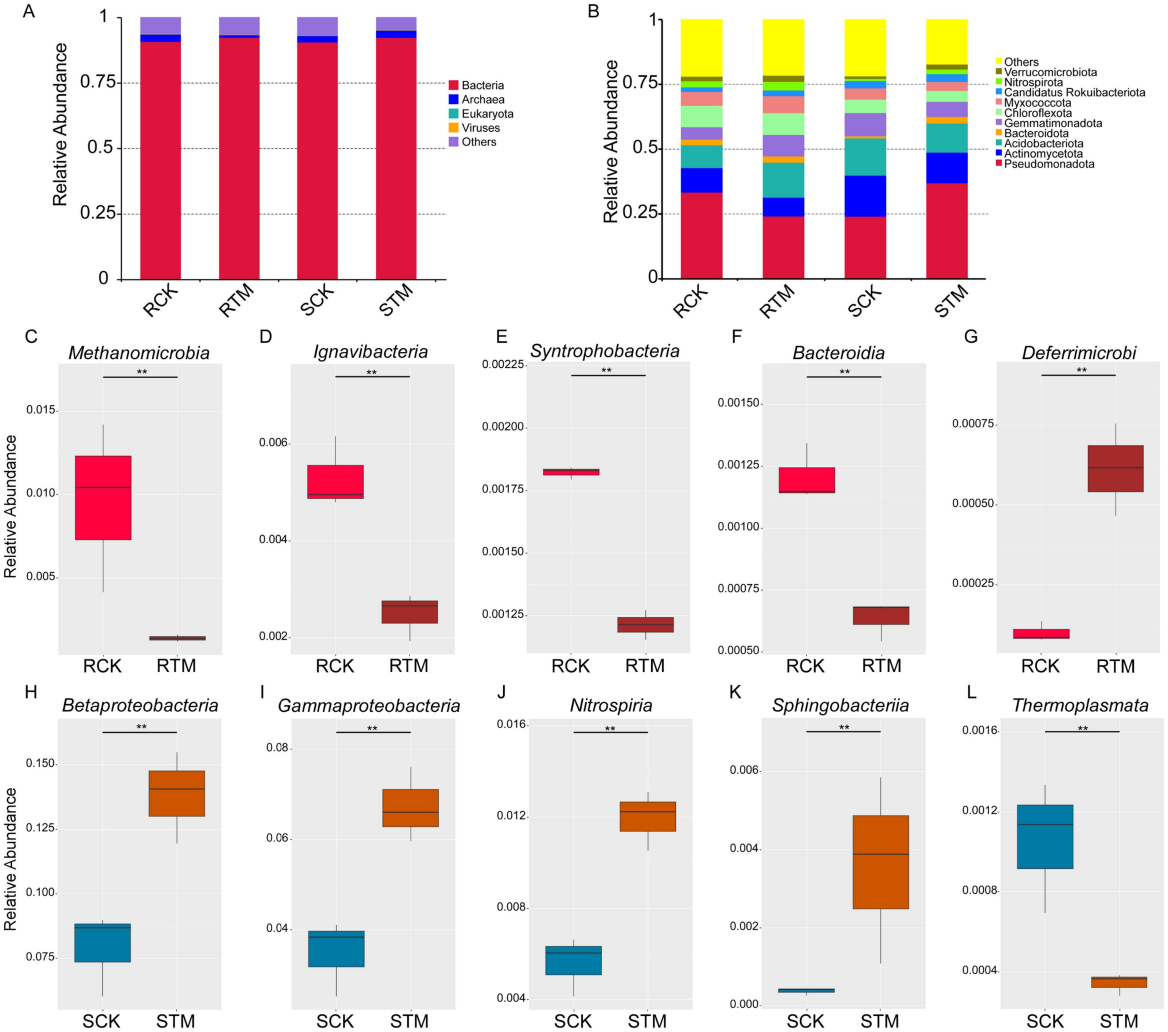
Analysis of soil microbiome composition in different plant soils of *Morchella sextelata*. Relative abundance of the top 10 most abundant soil microbiota in paddy and sandy soils at the **(A)** kingdom and **(B)** phylum levels. Variation in the top five most abundant microbial classes analyzed using metagenome sequencing (MetaGenomeSeq) during *M. sextelata* cultivation in **(C–G)** paddy soil and **(H–L)** sandy soil.

To further investigate the differences between paddy and sandy soils, we performed metagenomic sequencing to analyze soil microbiome composition. Metagenomic sequencing of the 12 soil samples yielded 165.78 GB of high-quality data (Supplementary Table S1), enabling a comprehensive profiling of the microbial communities. Taxonomic annotation identified a vast diversity across four kingdoms to 31,737 species (Supplementary Table S2), with bacteria constituting the dominant domain. Notably, the relative abundance of bacteria was further enhanced following *M. sextelata* inoculation (Figure 3A). In contrast, the abundances of viruses and archaea exhibited opposite responses to cultivation, decreasing in paddy soil but increasing in sandy soil. At the phylum level, the most abundant bacterial lineages in the cultivated soils included Verrucomicrobiota, Nitrospirota, Candidatus Rokuibacteriota, Myxococcota, Chloroflexota, Gemmatimonadota, Bacteroidota, Acidobacteriota, Actinomycetota, and Pseudomonadota (Figure 3B).

To pinpoint specific taxa driving the differences between soil types and cultivation states, we performed MetaGenomeSeq analysis. This revealed distinct differential taxa, *Methanomicrobia*, *Ignavibacteria*, *Syntrophobacteria*, and *Bacteroidia* were significantly decreased in paddy soil, while *Candidatus Deferrimicrobia* was increased (Figures 3C–G). Conversely, *Betaproteobacteria*, *Gammaproteobacteria*, *Nitrospiria*, and *Sphingobacteriia* were enriched in sandy soil, while *Thermoplasmata* was depleted (Figures 3H–L). With the exception of the archaeal classes *Methanomicrobia* and *Thermoplasmata*, all these differentially abundant taxa belonged to bacteria. Taken together, beyond confirming the fundamental compositional divergence between paddy and sandy soils, our metagenomic findings illuminate how *M. sextelata* cultivation exerts contrasting effects on the soil microbiota. These effects are evident in the opposing trajectories of bacterial, archaeal, and viral abundances, and are further defined by the enrichment or depletion of distinct bacterial classes with putative roles in nutrient cycling.

### 3.3 Soil microbial community diversity of alpha diversity and LEfSe analysis

**Figure 4:**
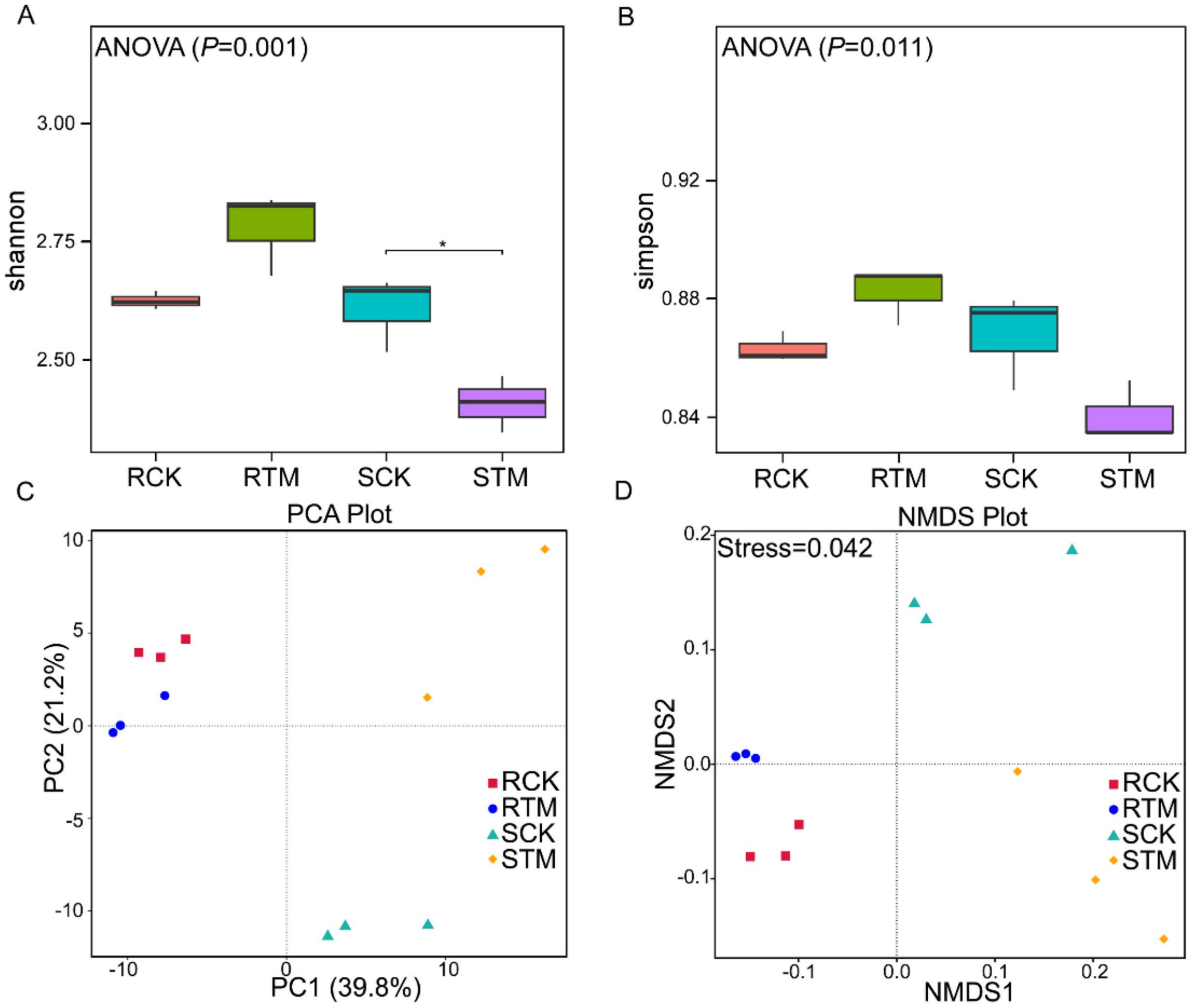
Variation in soil microbiome composition between paddy soil and sandy soil during *Morchella sextelata* cultivation. **(A,B)** Alpha diversity of the microbial community based on Shannon’s index and Simpson’s index. Data were analyzed using one-way analysis of variance (ANOVA); ^*^p < 0.05. **(C,D)** Principal component analysis (PCA) and nonmetric multidimensional scaling (NMDS) of the microbial community at the class level.

To analyze the microbial community diversity in *M. sextelata*-cultivated soils, we performed alpha diversity analysis on the soil samples. Our results showed that microbial alpha diversity differed significantly between paddy and sandy soils (Figures 4A,B). Shannon’s and Simpson’s index values were higher in RTM than RCK, indicating an increase in the species diversity and evenness of the microbial community in paddy soil after *M. sextelata* cultivation. In contrast, Shannon’s and Simpson’s index values were lower in STM compared to SCK, suggesting a decrease in microbial diversity in sandy soil following cultivation. Principal coordinates analysis and nonmetric multidimensional scaling (NMDS) analyses further corroborated these differences (Figures 4C,D) (Supplementary Tables S3, S4). The analysis revealed that the RCK and RTM in paddy soil clustered closely together, indicating that cultivation did not induce drastic changes in the overall structure of its microbial community. In stark contrast, the SCK and STM in sandy soil exhibited clear separation, demonstrating that cultivation significantly reshaped the microbial community structure in this soil type. In summary, the impact of *M. sextelata* cultivation on the soil microbial community was highly dependent on the soil type.

**Figure 5:**
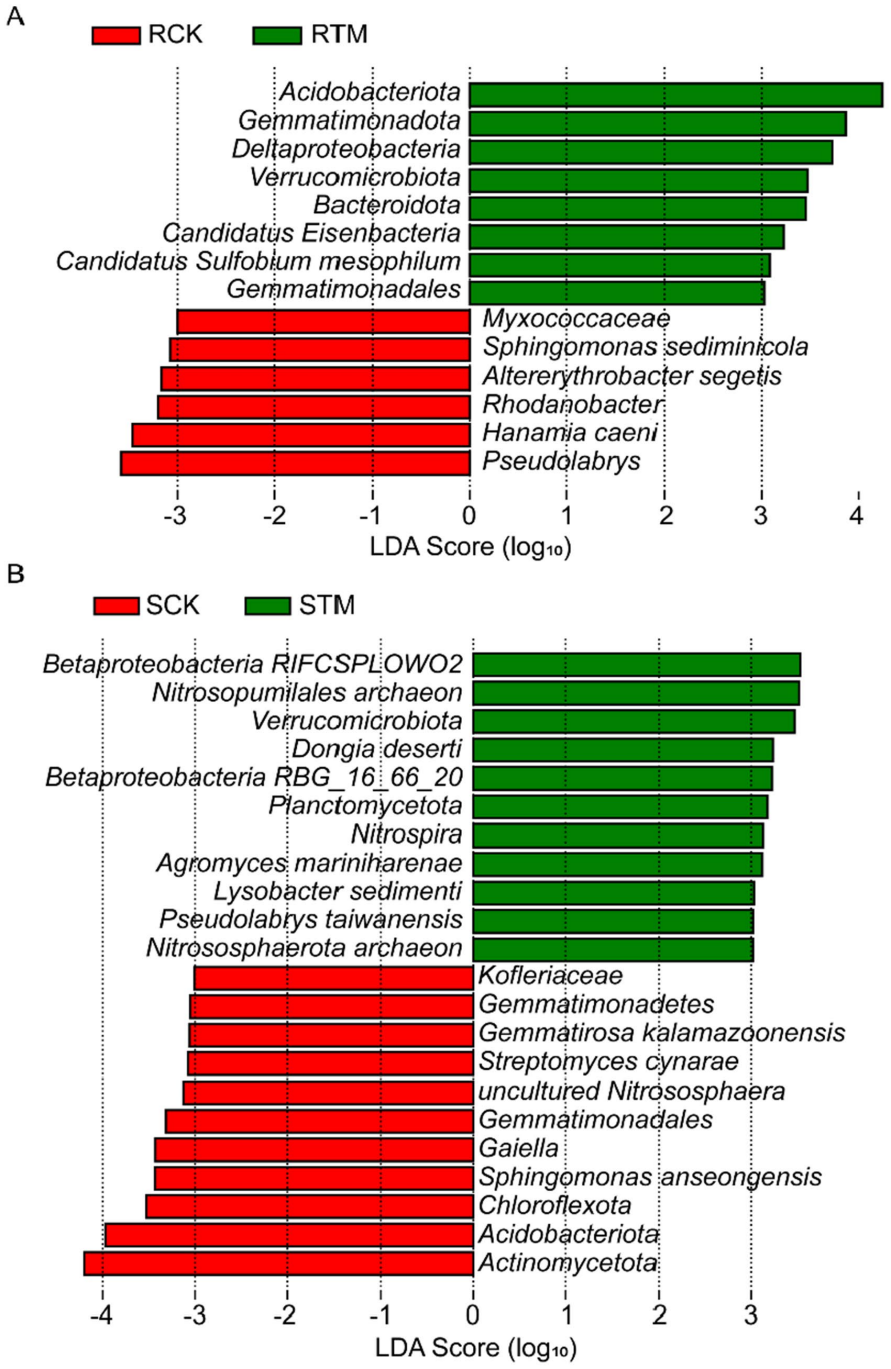
Linear discriminant analysis effect size (LEfSe) of key microbial biomarkers at the species level in paddy and sandy soils. **(A)** Paddy soil and **(B)** sandy soil. Only microbial taxa with a linear discriminant analysis (LDA) score >3 are shown.

To identify taxa with significant differences between paddy and sandy soils, we conducted LEfSe (Linear discriminant analysis Effect Size) analysis. The results revealed 14 biomarkers enriched in paddy soil, with *Acidobacteriota* and *Pseudolabrys* being the most abundant (Figure 5A). In contrast, 22 biomarkers were identified in sandy soil, with *Betaproteobacteria* and *Actinomycetota* showing the highest abundance (Figure 5B). The greater number of discriminant taxa in sandy soil suggests that the microbial community structure in this soil type was more distinctly altered or exhibited stronger specific responses compared to that in paddy soil.

### 3.4 Functional analysis of in *Morchella sextelata*-cultivation soil microbial gene

**Figure 6:**
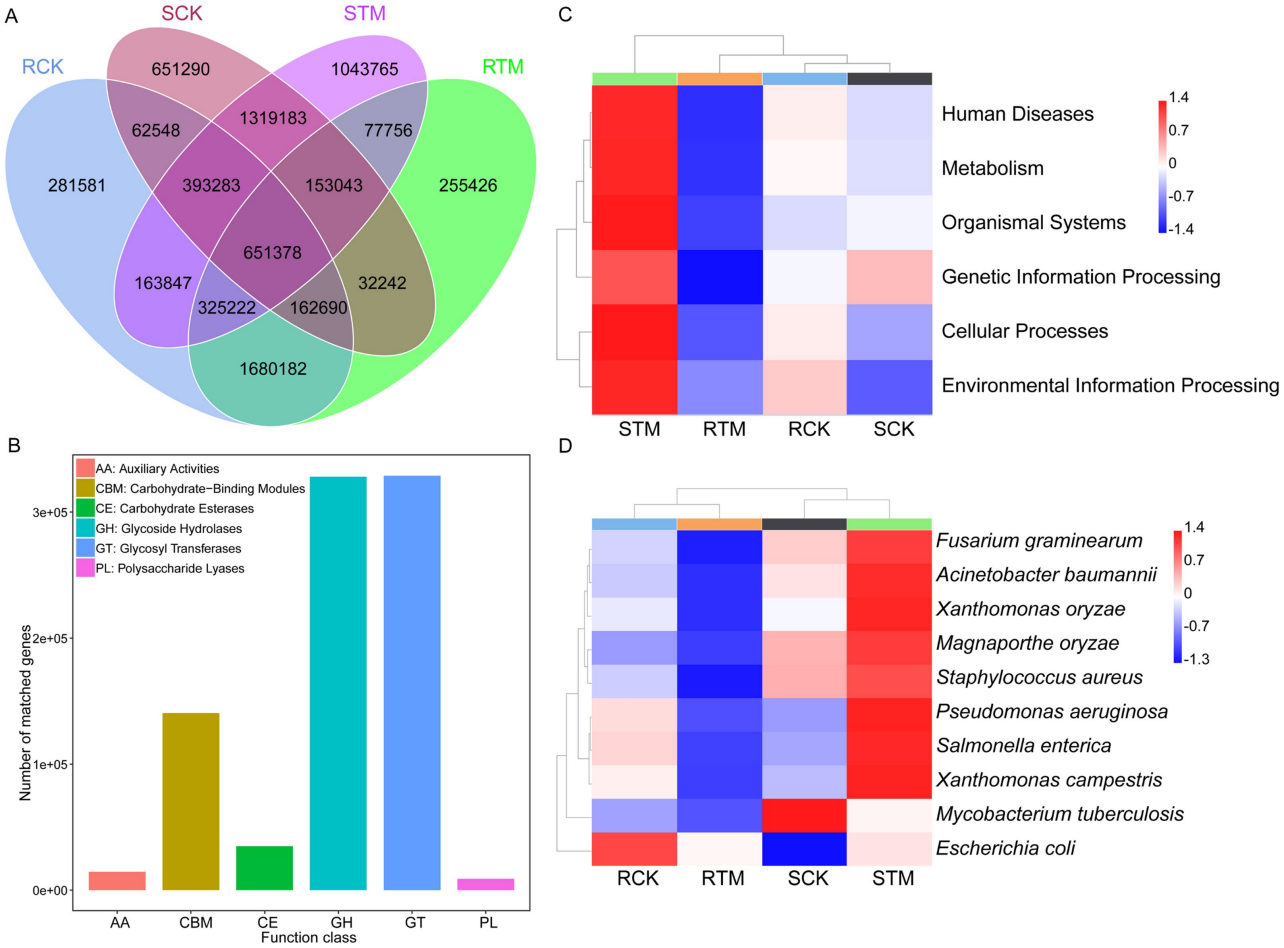
Functional analysis of soil microbiome genes. **(A)** Venn diagram of soil microbial gene profiles. **(B)** Functional annotation based on the Carbohydrate-Active enZymes (CAZy). **(C)** Kyoto Encyclopedia of Genes and Genomes (KEGG) enrichment analysis of soil microbial genes. **(D)** Pathogenicity analysis of the soil microbiome based on the Pathogen–Host Interactions (PHI) database.

To explore the biological functions of soil microbial genes, we conducted functional annotation using MetaGeneMark, the CAZy database, KEGG orthology, and the PHI database. Our results showed that sandy soil contained more microbial genes than paddy soil, with most genes enriched in glycoside hydrolases and glycosyl transferases, indicating that the carbohydrate metabolic pathways of soil microbes are highly active during morel cultivation (Figures 6A,B). Further functional analysis revealed that microbial genes in sandy soil were more enriched in pathways related to human diseases, metabolism, organismal systems, genetic information processing, cellular processes, and environmental information processing (Figure 6C). In contrast, the enrichment patterns in paddy soil showed the opposite trend. Additionally, PHI-based analysis showed that, except for *Mycobacterium tuberculosis*, the top 10 pathogens increased in abundance in sandy soil following *M. sextelata* cultivation, while they decreased in paddy soil (Figure 6D) (Supplementary Tables S5, S6). These findings suggest that *M. sextelata* cultivation may pose a higher disease risk in sandy soil.

## 4 Discussion

In this study, we conducted a comparative analysis of two common *M*. *sextelata* cultivation soils—paddy soil and sandy soil by assessing their physicochemical properties and microbial communities. The results highlight the distinct advantages and limitations of each soil type, offering a theoretical basis for optimizing *Morchella* cultivation practices.

Although ENB are essential in *Morchella* cultivation, soil remains the primary growth substrate, and its physicochemical properties closely associated to yield—particularly in relation to continuous cropping obstacles (Liu et al., 2018; Yuan et al., 2021). Consistent with previous studies, nitrogen and phosphorus levels increased in sandy soil following morel cultivation (Tan et al., 2019; Tan et al., 2021b). As a saprophytic fungus, *Morchella* naturally enriches soil organic carbon, nitrogen, and phosphorus during its growth. Paradoxically, such nutrient accumulation may inhibit primordia initiation and fruiting body development, thereby adversely affecting yield. In contrast, paddy soil showed no significant nutrient buildup, with nitrogen and phosphorus levels remaining stable, suggesting its potential in mitigating nutrient-related cultivation challenges (Figures 1, 2).

Unlike nitrogen and phosphorus, potassium plays a vital role in *Morchella* production, serving as a key nutrient for mycelial growth and primordia formation (Li et al., 2017). This is supported by the common practice of amending soil with potassium-rich plant ash to enhance yield, a strategy corroborated by the significant potassium depletion observed during cultivation in this study (Liu et al., 2023a). Additionally, trace elements such as Fe, Zn, and Mn, along with their compounds, may also affect yield outcomes (Liu et al., 2017). A notable limitation of this study, however, is the lack of investigation into the presence of these elements in both paddy and sandy soils.

There is a direct shaping influence of soil’s physicochemical properties on its microbial community structure. As an edible fungus, *Morchella* cultivation is often associated with a noticeable decline in overall soil microbial diversity (Tan et al., 2021a). In our study, we observed divergent trends between the two soil types. In paddy soil, total microbial diversity increased slightly after cultivation. In contrast, sandy soil exhibited a significant decline in overall microbial diversity (Figures 4A,B). This pattern in paddy soil aligns with previous reports of substantial reductions in bacterial diversity following *Morchella* cultivation (Orlofsky et al., 2021; Yu et al., 2022). However, a distinct phenomenon was identified in sandy soil: while total microbial diversity declined, the specific component of bacterial diversity increased markedly. This increase was driven by a significant rise in specific bacterial taxa (e.g., *Betaproteobacteria* and *Gammaproteobacteria*), likely due to elevated nutrient concentrations after cultivation (Figures 3H,K) (Falkowski et al., 2008). Notably, the rapid proliferation of such copiotroph bacteria may disrupt the ecological balance of microbial networks by competitively excluding other functional groups, thereby potentially exacerbating the instability of the soil micro-ecosystem.

Previous studies have shown that acidic metabolites produced during *Morchella* growth led to an increase in soil pH, which paradoxically reduces the substrate’s, thereby raising the risk of disease during cultivation (Li et al., 2023; Yin et al., 2025). Metagenomic analysis of PHI-database also revealed a significant reduction in disease-related genes in paddy soil, while a sharp increase was observed in sandy soil (Figure 6). In morel cultivation, many pathogens were enriched in soil with the continuous cropping. *Fusarium*, which has more species in sandy soil than paddy soil, caused many rots disease in field (Supplemental Table S7) (Masaphy, 2022; Zhu et al., 2023; Xie J. et al., 2024). Moreover, after morel cultivation, many crop diseases pathogen reduced in paddy soil, indicating that *Morchella* cultivation in paddy soil can effectively alleviate continuous cropping obstacles. Therefore, paddy soil for morel cultivation could reduce the accumulation and occurrence of diseases.

## Data Availability

The datasets presented in this study can be found in online repositories. The names of the repository/repositories and accession number(s) can be found at: https://www.ncbi.nlm.nih.gov/, PRJNA1305627.
